# Objective evaluation of size and shape of superficial foveal avascular zone in normal subjects by optical coherence tomography angiography

**DOI:** 10.1038/s41598-018-28530-7

**Published:** 2018-07-04

**Authors:** Hideki Shiihara, Hiroto Terasaki, Shozo Sonoda, Naoko Kakiuchi, Yuki Shinohara, Masatoshi Tomita, Taiji Sakamoto

**Affiliations:** Department of Ophthalmology, Kagoshima University Graduate School of Medical and Dental Sciences, Kagoshima, Japan

## Abstract

This study was conducted to investigate the size and shape of the foveal avascular zone (FAZ) determined by optical coherence tomography angiography (OCTA) and the relationship of the size and shape to the clinical findings in normal subjects. This was a cross-sectional study with seventy eyes of 70 volunteers. The size of the superficial FAZs were assessed by its area, length of perimeter, and Feret’s diameter, and the shape by the circularity, axial ratio, roundness, and solidity. The correlations between each parameter and the clinical findings were statistically determined. The coefficients of variation (CV) of the parameters of FAZ size were higher than that of the parameters of FAZ shape. The refractive error and axial length were significantly correlated with area-related factors. The central macular thickness (CMT) was significantly correlated with all parameters. Although the CMT was a critical factor that was significantly correlated with the size and shape characteristics of the FAZ, the shape might be a better factor for characterizing the FAZ than the size because of the low CV of shape-related factors and the characteristics are less affected by the other ocular factors.

## Introduction

Fluorescein angiography (FA) and indocyanine green angiography (ICGA) have been important methods to evaluate retinal diseases^1–3^. However, there are some drawbacks of FA and ICGA one of which is the need to use intravenous dye injections^4,5^.

Optical coherence tomography angiography (OCTA) is a newly developed method that is noninvasive and does not require the intravenous injection of contrast dyes. It is based on motion contrast imaging of high-resolution volumetric blood flow that can generate *en face* angiographic images of the capillary plexuses in the different layers of the retina and choroid in seconds^6^. OCTA has become widely used because of its less-invasiveness, and its ability to detect retinal microcapillaries in different layers of the retina with volume rendering which is not possible by FA or ICGA^7^.

The foveal avascular zone (FAZ) is a region surrounding the fovea which is devoid of retinal capillaries. The role of the FAZ in vision has been investigated extensively, and it has been found that the size of the FAZ was closely related to vision and can be measured with good repeatability and reproducibility by FA^8^. However, the size of the FAZ determined by FA was less than 40% of that determined by OCTA which limits the usefulness of FA in determining the size of the FAZ^9^.

The size of the FAZ has been intensively studied. For example, the area of the FAZ detected by OCTA could be an indicator of retinal disorders such as diabetic retinopathy and retinal vascular occlusion^10–17^. Nonetheless, it is unclear whether the size of the FAZ is the single best indicator of the presence of retinal pathology or the effectiveness of a treatment protocol because it has relatively large variability^18–22^.

Recently, the shape of the FAZ in the OCTA image has been demonstrated to be a good indicator of retinal pathology. The circularity and axial ratio of the FAZ in eyes with diabetic retinopathy were significantly different from that in normal eyes^23,24^, and the circularity of the FAZ was significantly lower in glaucomatous eyes with central visual field defects than in patients with peripheral visual field defects^25^. Although it was reported that the area of the FAZ was significantly correlated with the age, sex, axial length, refractive error, and the central macular thickness (CMT)^19–22,26–29^, there are only a few reports about the shape of the FAZ in normal eyes^30^. More importantly, to the best of our knowledge, there is no report on the relationship between the shape of the FAZ and the other ocular parameters in normal eyes. To diagnose and follow a diseases process accurately, it is essential to know the variations in both the size and shape of the FAZ in normal eyes.

Thus, the aim of this study was to quantify the size and shape of the FAZ in normal eyes. To accomplish this, we analyzed the OCTA images of 73 volunteers with right eyes. In addition to the regular parameters of the size of the FAZ, we also analyzed different measures of the shape of the FAZ.

## Results

Two cases were excluded due to poor image quality and one case due to the presence of an epiretinal membrane. In the end, we studied 70 volunteers (38 men and 32 women) whose average ± SD age was 35.5 ± 9.6 years with a range of 20 to 61 years. The average refractive error was −2.2 ± 1.8 diopters (D) with a range of +0.875 to −5.75 D. The average axial length was 24.5 ± 1.2 mm with a range of 21.1 to 27.1 mm. The average CMT was 240.4 ± 19.6 μm with a range of 200 to 286 μm.

### Repeatability of values of each parameter in same eye

The intra-rater correlation coefficient was >0.916 for each of the measures (Table 1). The inter-rater correlation coefficient was more than 0.876 for each of the measures which was also very high (Table 1).

**Table 1:** Intra-rater correlation coefficient and Inter-rater correlation coefficient of each parameter.

	Intra-rater correlation coefficient	Inter-rater correlation coefficient
	ICC (95%CI)	ICC (95%CI)
Area	0.998 (0.995–0.999)	0.984 (0.240–0.992)
Perimeter	0.995 (0.989–0.997)	0.979 (0.694–0.994)
Feret’s diameter	0.993 (0.986–0.997)	0.975 (0.849–0.992)
Circularity	0.943 (0.885–0.972)	0.918 (0.836–0.960)
Axial ratio	0.964 (0.926–0.982)	0.945 (0.888–0.973
Roundness	0.964 (0.926–0.983)	0.939 (0.876–0.970)
Solidity	0.916 (0.832–0.959)	0.876 (0.758–0.939)

### Parameters related to size of the FAZ

The average (±SD) FAZ area was 0.329 ± 0.115 mm^2^, the average length of the perimeter was 2.279 ± 0.418 mm, and the average Feret’s diameter was 0.749 ± 0.129 mm.

### Parameters related to shape of the FAZ

The average (±SD) circularity was 0.769 ± 0.064, the average axial ratio was 1.147 ± 0.103, the average roundness was 0.878 ± 0.071, and the average solidity was 0.925 ± 0.029.

### Coefficients of variation

**Figure 1:**
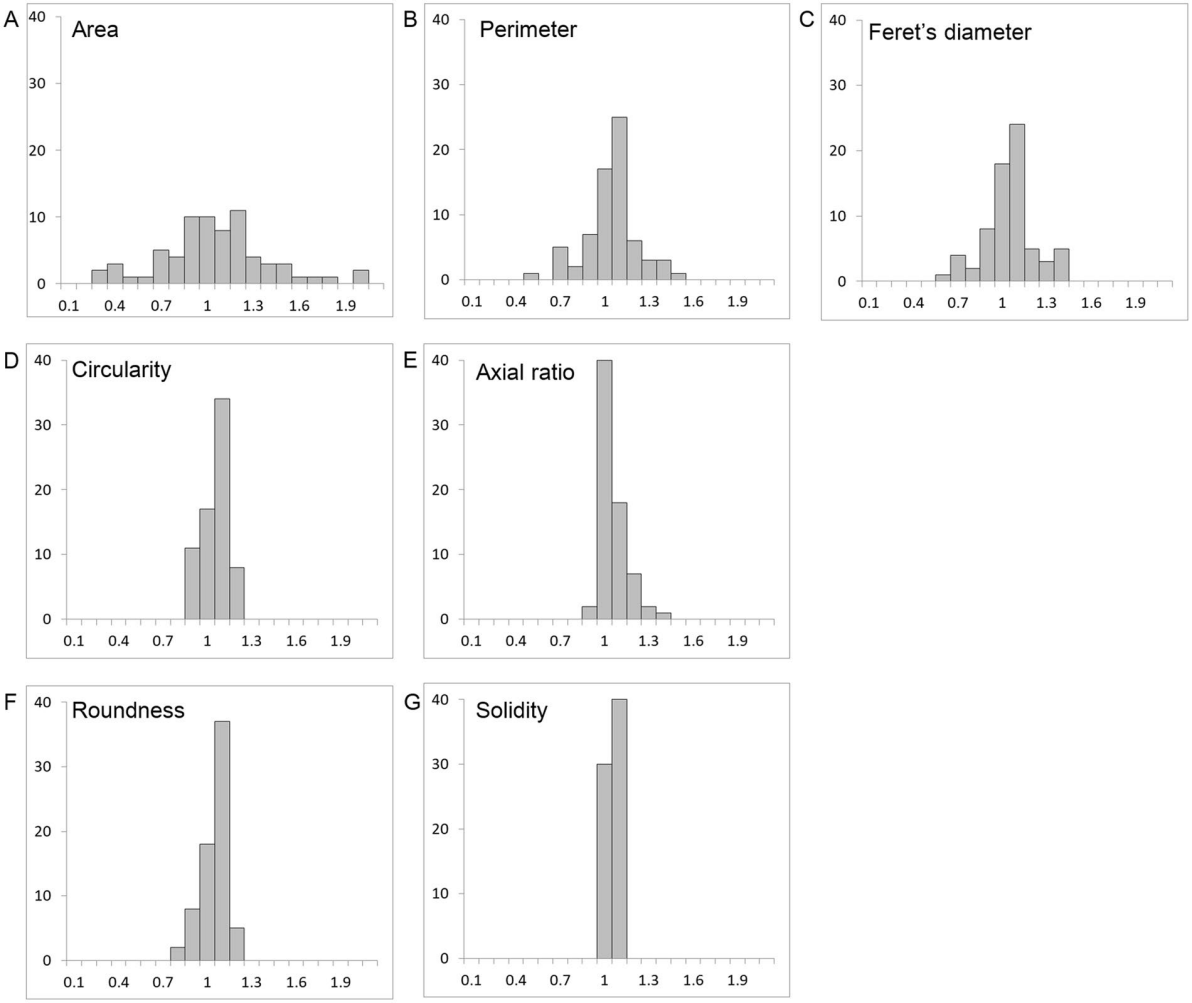
Histograms normalized by the average of each parameter. The coefficient of variation of the parameters related to the size of the FAZ, viz., the area (**A**), perimeter (**B**) and Feret’s diameter (**C**), were larger than that for the parameters associated with the shape of the FAZ, viz., circularity (**D**), axial ratio (**E**), roundness (**F**), and solidity (**G**).

The coefficients of variation of the size of the FAZ were; area was 0.35, perimeter was 0.18, and Feret’s diameter was 0.17, while those of the shape of the FAZ were; circularity was 0.08, axial ratio was 0.09, roundness was 0.08, and solidity was 0.03 (Table 2). The histograms that were normalized by the average of each parameter (Fig. 1) had a smaller range of the coefficients of variation for the parameters related to the shape of the FAZ than that in parameters related to the size of the FAZ.

**Table 2:** Parameters of the foveal avascular zone.

	Average ± SD	CV	Range
Area, mm^2^	0.329 ± 0.115	0.35	0.073 to 0.656
Perimeter, mm	2.279 ± 0.418	0.18	1.115 to 3.347
Feret’s diameter, mm	0.749 ± 0.129	0.17	0.375 to 1.047
Circularity	0.769 ± 0.064	0.08	0.617 to 0.900
Axial ratio	1.147 ± 0.103	0.09	1.015 to 1.516
Roundness	0.878 ± 0.071	0.08	0.660 to 0.985
Solidity	0.925 ± 0.029	0.03	0.834 to 0.972

### Factors affecting values of the parameters

Next, we examined whether the sex and other ocular parameters affected the size and shape of the FAZ. Our analysis showed that the FAZ area, perimeter, and Feret’s diameter were larger in women than men (*P* < 0.001, for each. Table 3). The circularity, axial ratio, roundness, and solidity of the FAZ were not significantly different between women and men. Pearson’s correlational coefficient showed that age was not significantly correlated with any of the examined parameters. The refractive error was significantly correlated with the Feret’s diameter (*P* = 0.043). The axial length was significantly correlated with the FAZ area (*P* = 0.01), the perimeter (*P* = 0.01), and Feret’s diameter (*P* < 0.001).

**Table 3:** Difference of sex on the each parameter.

	Male (n = 38)	Female (n = 32)	P value
Area, mm^2^	0.274 ± 0.097	0.390 ± 0.106	**<0.001**
Perimeter, mm	2.091 ± 0.399	2.490 ± 0.342	**<0.001**
Feret’s diameter, mm	0.688 ± 0.120	0.818 ± 0.105	**<0.001**
Circularity	0.760 ± 0.067	0.780 ± 0.061	0.204
Axial ratio	1.160 ± 0.127	1.132 ± 0.067	0.266
Roundness	0.871 ± 0.086	0.886 ± 0.052	0.390
Solidity	0.919 ± 0.030	0.932 ± 0.257	0.054

The CMT was significantly correlated with the area, perimeter, and Feret’s diameter (*P* < 0.001) of the size of the FAZ and also with the circularity (*P* = 0.11), axial ratio (*P* = 0.012), roundness (*P* = 0.022), and solidity (*P* = 0.001) of the FAZ (Table 4).

**Table 4:** Correlation of clinical findings and each parameter.

	Area		Perimeter		Feret’s diameter		Circularity		Axial ratio		Roundness		Solidity	
	R	P value	R	P value	R	P value	R	P value	R	P value	R	P value	R	P value
Age	0.060	0.620	0.099	0.413	0.120	0.321	−0.178	0.140	0.155	0.200	−0.171	0.157	−0.163	0.179
Spherical equivalent, D	0.212	0.078	0.210	0.081	0.242	**0.043**	0.120	0.324	0.129	0.286	−0.158	0.193	0.175	0.148
Axial length, mm	−0.395	**0.001**	−0.395	**0.001**	−0.424	**<0.001**	−0.075	0.539	0.015	0.903	0.024	0.845	−0.165	0.172
Central macular thickness, μm	−0.756	**<0.001**	−0.704	**<0.001**	−0.708	**<0.001**	−0.304	**0.011**	0.299	**0.012**	−0.272	**0.022**	−0.380	**0.001**

**Figure 2:**
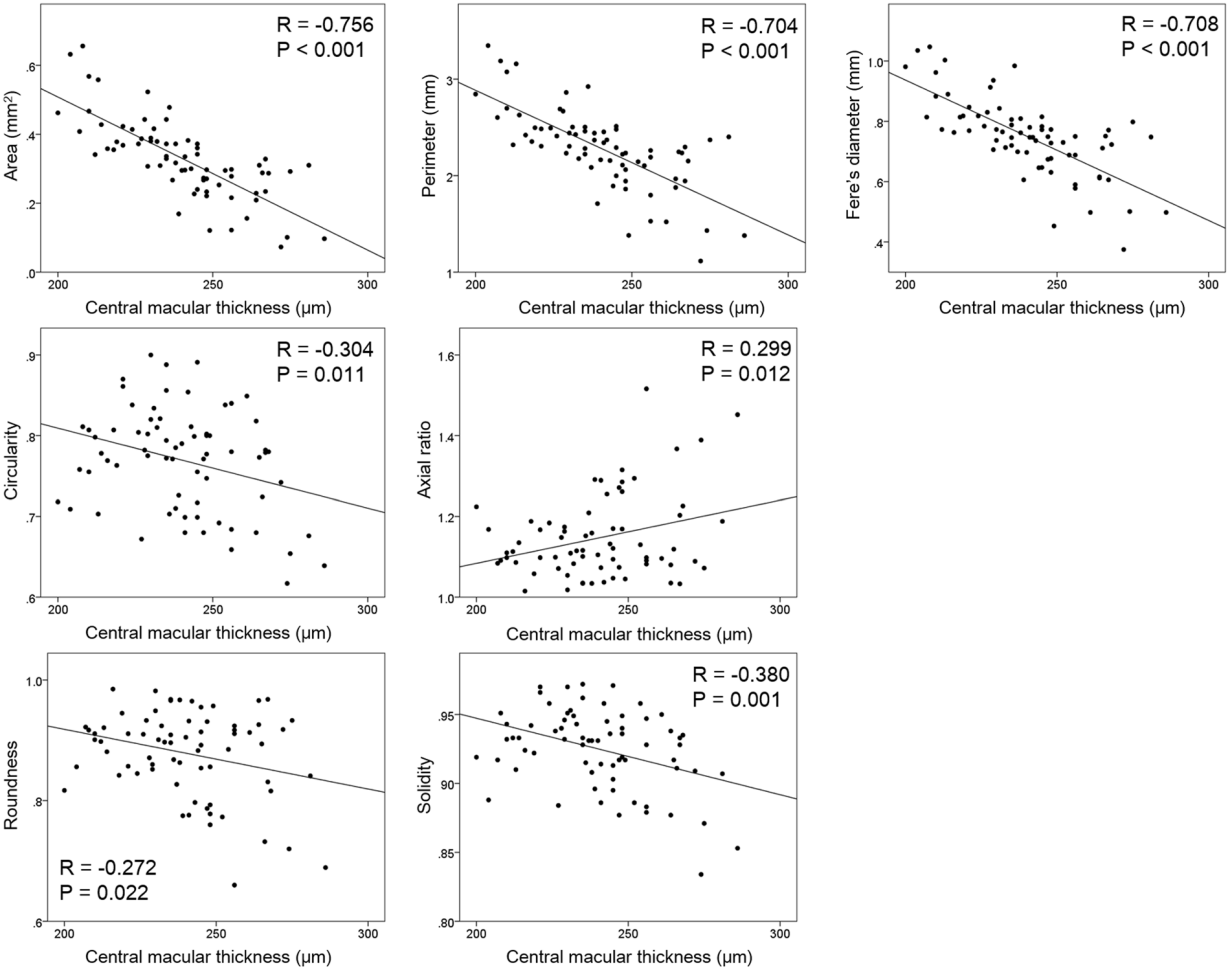
Correlation between the central macular thickness and each parameter. All parameters were significantly correlated with the central macular thickness.

Multiple linear analysis showed that axial length was significantly correlated with the area, perimeter, and Feret’s diameter of the size characteristics of the FAZ (*P* < 0.001, for each). The CMT was significantly correlated with the area, perimeter, and Feret’s diameter of the FAZ (*P* < 0.001), and also with the circularity (*P* = 0.011), axial ratio (*P* = 0.012), roundness (*P* = 0.022), and solidity (*P* = 0.001; Table 5). The correlations between CMT and each parameter are shown in Fig. 2.

**Table 5:** Multiple linear regression analysis of factors affecting each parameter.

	Area		Perimeter		Feret’s diameter		Circularity		Axial ratio		Roundness		Solidity	
	R	P value	R	P value	R	P value	R	P value	R	P value	R	P value	R	P value
	*R*^2^ = 0.651		*R*^2^ = 0.588		*R*^2^ = 0.632		*R*^2^ = 0.092		*R*^2^ = 0.089		*R*^2^ = 0.074		*R*^2^ = 0.144	
Sex	—	—	—	—	—	—	—	—	—	—	—	—	—	—
Age	—	—	—	—	0.172	**0.026**	—	—	—	—	—	—	—	—
Spherical equivalent, D	—	—	—	—	—	—	—	—	—	—	—	—	—	—
Axial length, mm	−0.287	**<0.001**	−0.308	**<0.001**	−0.302	**<0.001**	—	—	—	—	—	—	—	—
Central macular thickness, μm	−0.712	**<0.001**	−0.657	**<0.001**	−0.683	**<0.001**	−0.304	**0.011**	0.299	**0.012**	−0.272	**0.022**	−0.380	**0.001**

## Discussion

The results showed that the parameters related to the size of the FAZ had greater variations than those related to the shape of the FAZ. The size of the FAZ area ranged from 0.073 mm^2^ to 0.656 mm^2^, and the coefficient of variation was 0.35. The coefficient of variation was 0.18 for the length of perimeter and 0.17 for the Feret’s diameter. On the other hand, the coefficient of variation for the four morphological parameters, viz., the circularity, axial ratio, roundness, and solidity varied between 0.03 and 0.09, which are significantly smaller than those related to the size of the FAZ. Given that the morphological parameters of the FAZ are less affected by individual variations than the size of the FAZ in normal eyes, it is possible that the parameters related to the shape of the FAZ may be better parameters for monitoring the FAZ in disease suspects. Indeed, the circularity and axial ratio are changed significantly more in in eyes with diabetic retinopathy than the size of the FAZ^23,24^.

We and another group demonstrated that the FAZ area of the same eye was different depending on the OCT device used to obtain the size^31,32^. Thus, it would not be appropriate to use the area obtained by difference OCT devices as a parameter of the FAZ in clinical trials. The multiple linear regression analysis showed that parameters related to the FAZ size were significantly affected by the axial length, however the shape of the FAZ was not correlated. These findings indicate that the shape of the FAZ may be a better parameter to assess the FAZ. This is especially important in large-scale studies where multiple OCTA instruments are used. If the shape parameters are proven to be interchangeable between machines, they will become much better than the size parameters of the FAZ to assess the eyes of disease suspects.

There have been many studies on the factors related to the size of the FAZ^19–22,26–29^. Our results showed that the parameters tended to be larger in women than men, in eyes with shorter axial lengths, and in eyes with a thinner CMT. These findings are consistent with the earlier reports^26–29^. The relationship between the size of FAZ and age is still inconclusive, some showed significant relationship^27,29^, while others showed insignificant^11,26,28^. In this study, the multiple regression analysis showed only Feret’s diameter was significantly increased with age. The difference among studies might be due to the large variation of FAZ size.

**Figure 3:**
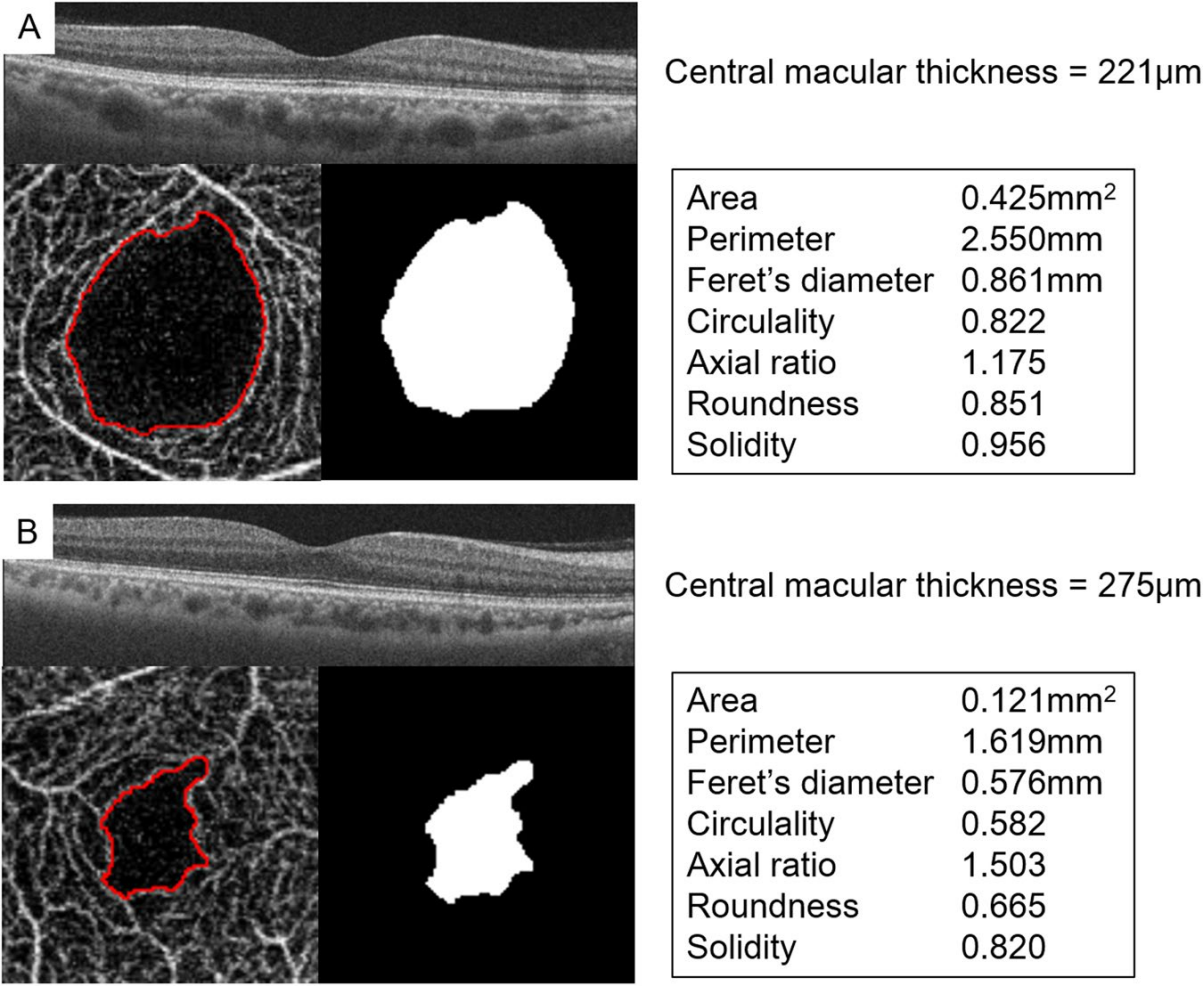
Characteristics of the shape of the FAZ in an eye with a thin fovea and an eye with a thick fovea. (**A**) Thin fovea had larger size of FAZ and circular-shaped FAZ. (**B**) Thick fovea had smaller size of FAZ and irregular-shaped FAZ.

To the best of our knowledge, there has not been any studies that investigated the factors affecting the parameters related to the shape of the FAZ. Our results showed that the sex, age, and refractive error were not significantly correlated with any of the shape parameters. Only the CMT was found to be significantly correlated with the shape of the FAZ (Table 4). Thus, the retinas with thick CMT had smaller size the FAZ and more irregular shaped the FAZ, which is supported by the present results of lower values of circularity, roundness, solidity, and higher axial ratio (Fig. 3).

The CMT has been reported to be correlated with the age, sex, spherical equivalent, axial length, ethnicity, BMI, smoking, and gestational age at birth although the underlying mechanism for the differences in the CMT has not been determined^33–35^. The variations in the size of the FAZ and CMT can be explained by the process of foveal development. A study of the development of the primate fovea suggest that the organization of the foveal layers is influenced by the size of the FAZ and intraocular pressure^36^. Once the FAZ is formed at about 25-weeks-of-gestation, the foveal pit deepens progressively by intraocular pressure. Then, with the anterior-posterior axial growth, the inner retinal layers migrate away from the foveal center, the cone photoreceptors migrate into the foveal center, and the photoreceptors elongate^37,38^. Importantly, during the process migration of the astrocytes, the capillaries also shift outward from the fovea because they are attached to the astrocytes^39^. This results in the roundish shape of the FAZ.

A thick fovea is associated with a more compact inner retina, continuity of the inner nuclear layer (INL), and a smaller FAZ size, whereas a thin fovea is characterized by less compact inner retinal layers, discontinuity of the INL, and a larger FAZ area. In compact tissues, the FAZ border can be easily deformed into an irregular shape which may explain the present finding that irregular shaped FAZs are associated with a thick fovea. It is known that preterm children have thick CMT and small FAZ area because the migration of the cells is incomplete or immature^34,35,40^. This phenomenon which can affect the CMT might also affect the shape of the FAZ even normal subjects with normal visual acuity.

There are limitations in this study. The Japanese population is known to be the most myopic population in the world^41^. As a result, most of the subjects were myopic which might have affected the results. On the other hand, the reliability of the examination was very high because no pathological factors such as cataract or vitreous opacities were present in the young healthy individuals. Second, this study examined only the superficial FAZ and not the deep FAZ which was not as clearly imaged as the superficial FAZ^32^. It has been reported that area of the superficial FAZ was well correlated with area of the FAZ from the whole retinal layer^28^. However, it should be remembered that the FAZ is the result of anatomical fusion of all the capillary layers within the inner retina. Therefore, any segmentation of the foveal and perifoveal capillaries can be an artifact of the current segmentation algorithm. It is necessary to develop another segmentation scheme for the FAZ that appropriately accounts for the unique anatomy of its neurosensory and vascular tissue. This is not a negligible limitation of current research studies using OCTA.

In conclusion, the variance in the shape parameters of the FAZ was smaller than that for the size of the FAZ based on OCTA analyses. Because the shape parameters had good repeatability and reproducibility in normal subjects, they are probably better parameters to evaluate the FAZ. Importantly, the CMT is a critical factor that is correlated with the size and shape of the FAZ. This should be remembered in the analysis of the FAZ.

## Methods

This was a prospective, cross sectional study that was performed at the Kagoshima University Hospital, Kagoshima, Japan, from May 2017 to June 2017. The procedures used in this study were approved by the Ethics Committee of Kagoshima University Hospital and registered with the University Hospital Medical Network (UMIN)-clinical trials registry (CTR). The registration title was, “UMIN000012310, Choroidal structure on OCT images for healthy eyes”. A detailed protocol is available at, http://www.kufm.kagoshima-u.ac.jp/~op/gairai/RCstructurestudy.html. A written informed consent was obtained from all the subjects after an explanation of the procedures to be used and possible complications, and all of the investigative procedures conformed to the tenets of the Declaration of Helsinki.

Seventy-three volunteers were initially enrolled, and all had a complete ocular examination including measurements of the spherical equivalent with an autorefractor/keratometer (RM8900; Topcon, Tokyo, Japan), best-corrected visual acuity (BCVA), and IOP with a pneumotonometer (CT-80; Topcon, Tokyo, Japan). They were also examined by slit-lamp biomicroscopy and ophthamoscopy. The axial length was measured by optical interferometry (OA-1000; Tomey, Tokyo, Japan).

The inclusion criteria were; age >20 years and <65 years, BCVA of ≥20/20, and normal fundus by ophthalmoscopy and OCT. The exclusion criteria were history of ocular and systemic diseases, prior ocular surgery or intraocular injections, and high myopia of ≤−6.0 diopters (D). Images with poor signal strength or with motion artifacts enough to disrupt a clear delineation of the FAZ were also excluded.

### Imaging protocol

OCTA images were obtained with a swept-source OCT device (DRI OCT Triton; Topcon, Tokyo, Japan) with a central wavelength of 1050 nm, an acquisition speed of 100,000 A-scans/sec, an axial resolution of 7 µm and a transverse resolution of 20 μm. Scans were 3 × 3 mm cubes with each cube consisting of 320 clusters of four repeated B-scans centered on the fovea. Based on these default settings, the superficial capillary network extended from 2.6 μm below the internal limiting membrane (ILM) to 15.6 μm below the inner plexiform layer (IPL). Because our preliminary results and other studies showed that the deep FAZ had less well-defined borders than the superficial FAZ, the present study was done only on the superficial FAZ.

To determine the central macular thickness (CMT), 7 × 7 mm cubes centered on the fovea was obtained by the swept-source OCT. A whole-retinal thickness map centered on the fovea was created using the Early Treatment Diabetic Retinopathy Study grid. The CMT was calculated by averaging the retinal thickness of the macula within 1 mm from fovea.

### Image analysis

**Figure 4:**
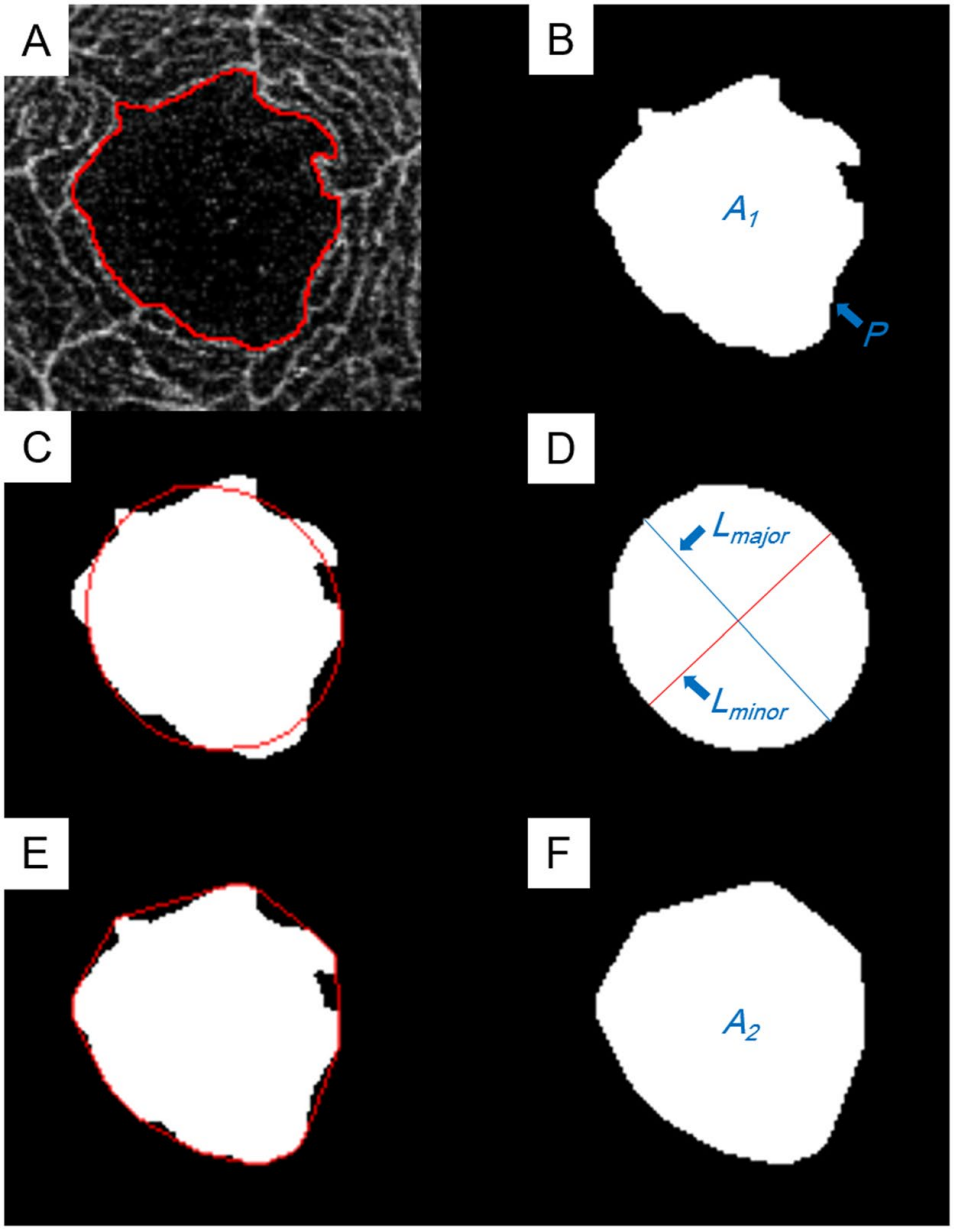
Parameters for assessing the foveal avascular zone (FAZ) obtained by optical coherence tomorgraphy angiography (OCTA). (**A**) The FAZ area was measured manually with the imageJ software. (**B**) Area (*A*_1_) and length of perimeter (*P*) was calculated automatically. Circularity was calculated as 4π × *A*_1_/*P*^2^. (**C**) Ellipse was semi-automatically fitted to the FAZ by imageJ. (**D**) The major axis (*L*_*major*_) and minor axis (*L*_*minor*_) lengths were automatically calculated. The axial ratio was calculated as *L*_*major*_/*L*_*minor*_. Roundness was calculated as 4π × A_1_/(*L*_*major*_)^2^. (**E**) The convex hull was semi-automatically fit by imageJ. (**F**) Area of convex square(*A*_2_) was automatically calculated. Solidity was calculated as *A1*/*A2*.

The OCTA images of the superficial capillary plexus were obtained as 312 × 312 pixel PNG images. The images were analyzed with the ImageJ software (ImageJ version 1.51, National Institutes of Health, Bethesda, MD; available at: http://imagej.nih.gov/ij/) to determine the size and shape of the FAZ. The FAZ was defined as the avascular region at the center of the fovea, and the border of the FAZ was manually drawn by a single retina specialist (HS) who was masked to the clinical information. The size of the FAZ was designated by the area (mm^2^), length of the perimeter (mm), and Feret’s diameter (mm), and the shape of the FAZ by the circularity, axial ratio, roundness, and solidity. The values of these parameters were calculated by ImageJ as reported^42^. (Fig. 4)

### Parameters related to size of the FAZ

The area, length of the perimeter, and Feret’s diameter of the FAZ were determined. The area and perimeter length were measured using the ImageJ software. Feret’s maximum diameter of the FAZ, also known as the maximum caliper, was measured with the ImageJ software.

### Parameters related to shape of the FAZ

The circularity, axial ratio, roundness, and solidity were determined to characterize the shape of the FAZ. The circularity is a shape descriptor that can mathematically indicate the degree of similarity of the FAZ to a perfect circle. A value of 1.0 designates a perfect circle, and as the circularity value decreases, the shape is increasingly less circular. Circularity is defined by the equation:

$${\rm{Circularity}}=4{\rm{\pi }}\times {\rm{area}}/{{\rm{perimeter}}}^{2}$$

The axial ratio is obtained from a best fit ellipse of the FAZ. The following parameters were determined from the best fit ellipse: the length of the major and minor axes and the axial ratio. The axial ratio is calculated by the following equation:

$${\rm{A}}{\rm{x}}{\rm{i}}{\rm{a}}{\rm{l}}\,{\rm{r}}{\rm{a}}{\rm{t}}{\rm{i}}{\rm{o}}=({\rm{l}}{\rm{e}}{\rm{n}}{\rm{g}}{\rm{t}}{\rm{h}}\,{\rm{o}}{\rm{f}}\,{\rm{m}}{\rm{a}}{\rm{j}}{\rm{o}}{\rm{r}}\,{\rm{a}}{\rm{x}}{\rm{i}}{\rm{s}})/({\rm{l}}{\rm{e}}{\rm{n}}{\rm{g}}{\rm{t}}{\rm{h}}\,{\rm{o}}{\rm{f}}\,{\rm{m}}{\rm{i}}{\rm{n}}{\rm{o}}{\rm{r}}\,{\rm{a}}{\rm{x}}{\rm{i}}{\rm{s}})$$

The roundness uses the best fit ellipse, and is similar to circularity but is not sensitive to irregular borders along the perimeter of the FAZ. Roundness is defined by the equation:

$${\rm{R}}{\rm{o}}{\rm{u}}{\rm{n}}{\rm{d}}{\rm{n}}{\rm{e}}{\rm{s}}{\rm{s}}=4\pi \times {\rm{a}}{\rm{r}}{\rm{e}}{\rm{a}}/{({\rm{l}}{\rm{e}}{\rm{n}}{\rm{g}}{\rm{t}}{\rm{h}}{\rm{o}}{\rm{f}}{\rm{m}}{\rm{a}}{\rm{j}}{\rm{o}}{\rm{r}}{\rm{a}}{\rm{x}}{\rm{i}}{\rm{s}})}^{2}$$

Solidity describes the extent to which a shape is convex or concave. The area enclosed by a convex hull can provide information regarding the solidity of the shape. The solidity of a completely convex shape is 1, the farther the solidity deviates from 1, the greater the extent of concavity in the structure. Solidity is defined by the equation:

$${\rm{Solidity}}={\rm{area}}/{\rm{convex}}\,{\rm{area}}$$

To assess the intra-rater agreement, one grader (HS) analyzed the same set of the FAZ a second time on another day. To assess the inter-rater agreement, two independent graders (HS and NK) identified the FAZ and calculated each parameter.

### Statistical analyses

All statistical analyses were performed with SPSS statistics 19 for Windows (SPSS Inc., IBM, Somers, New York, USA). The coefficient of variation (SD/mean) was calculated, and the intra-rater correlation coefficients were calculated using 1-way random effects model for measurements of agreement. The inter-rater correlation coefficients were calculated using 2-way mixed-effects model for measurements of absolute agreement. The significance of the differences between the two groups was determined by unpaired *t* tests, and bivariate relationships were analyzed using Pearson’s coefficients of correlation. A stepwise forward multivariate linear regression analysis was performed to evaluate the contribution of clinical findings to each morphologic parameter. A *P* value < 0.05 was taken to be statistically significant.
